# Correlating corneal arcus with atherosclerosis in familial hypercholesterolemia

**DOI:** 10.1186/1476-511X-7-7

**Published:** 2008-03-10

**Authors:** Loren A Zech, Jeffery M Hoeg

**Affiliations:** 1 Molecular Disease Branch, National Heart, Lung and Blood Institute, National Institutes of Health, Building 10/Room 7N115, 10 Center Drive MSC 1666, Bethesda, MD 20892, USA; 2 College of Medicine, University of Illinois at Urbana-Champaign, 190 Medical Sciences Building, 506 South Mathews Ave, Urbana, IL 61801, USA

## Abstract

**Background:**

A relationship between corneal arcus and atherosclerosis has long been suspected but is controversial. The homozygous familial hypercholesterolemia patients in this study present a unique opportunity to assess this issue. They have both advanced atherosclerosis and corneal arcus.

**Methods:**

This is a cross-sectional study of 17 patients homozygous for familial hypercholesterolemia presenting to the Clinical Center of the National Institutes of Health. Plasma lipoproteins, circumferential extent of arcus, thoracic aorta and coronary calcific atherosclerosis score, and Achilles tendon width were measured at the National Institutes of Health.

**Results:**

Patients with corneal arcus had higher scores for calcific atherosclerosis (mean 2865 compared to 412), cholesterol-year score (mean 11830 mg-yr/dl compared to 5707 mg-yr/dl), and Achilles tendon width (mean 2.54 cm compared to 1.41 cm) than those without. Corneal arcus and Achilles tendon width were strongly correlated and predictive of each other. Although corneal arcus was correlated with calcific atherosclerosis (r = 0.67; p = 0.004), it was not as highly correlated as was the Achilles tendon width (r = 0.855; p < 0.001).

**Conclusion:**

Corneal arcus reflects widespread tissue lipid deposition and is correlated with both calcific atherosclerosis and xanthomatosis in these patients. Patients with more severe arcus tend to have more severe calcific atherosclerosis. Corneal arcus is not as good an indicator of calcific atherosclerosis as Achilles tendon thickness, but its presence suggests increased atherosclerosis in these hypercholesterolemic patients.

## Background

The German pathologist Rudolf Virchow is credited with the hypothesis that atherosclerosis reflects insudation of pathogenic agents into tissue. He also noted, in 1852, the association of corneal arcus and atherosclerosis, and hypothesized a similar mechanism of formation[1]. In contrast, William Osler, in 1892, suggested that arcus senilis had little utility in diagnosing "fatty degeneration" of the heart [2]. The attempt to relate corneal lipid deposits and vascular lipid deposits has been and remains controversial, despite continued interest[3,4]. We investigate the relationship between corneal arcus (i.e. arcus senilis) and lipid deposition in other tissues in 17 patients homozygous for familial hypercholesterolemia (FH). This rare inborn error of metabolism is estimated to occur once in every million U.S. births [5]. However, the elucidation of the genetic basis of this metabolic disease has been central to understanding the role of particular lipoproteins in the pathogenesis of human atherosclerosis[6,7]. Quantifying the extent of arcus and other lipid deposits in patients with such profound hypercholesterolemia provides a means of assessing the clinical issues raised by Virchow and Osler.

**Figure 1:**
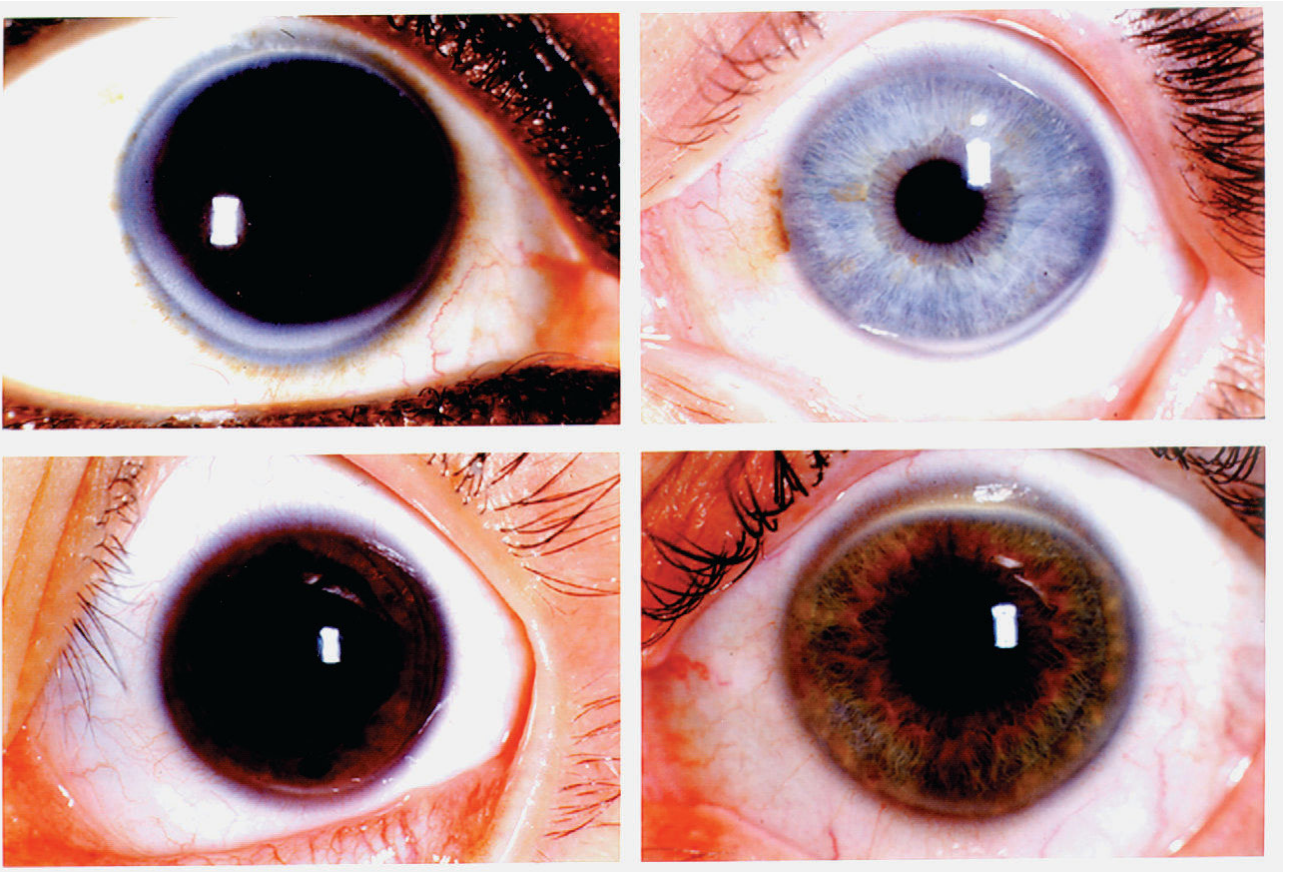
**Corneal arcus**. Four representative slides of corneal arcus. Arcus deposits tend to start at 6 and 12 o'clock and fill in until becoming completely circumferential. There is a thin clear section separating the arcus from the limbus known as the lucid interval of Vogt.

The deposition of lipid in the human cornea, macroscopically observed as corneal arcus (Fig. 1), is one of the classic physical stigmata of the FH homozygote, along with various types of xanthoma[5,8,9]. The predominant lipid in all these deposits is esterified cholesterol, the predominant lipid in low density lipoprotein (LDL) particles [10-17]. LDL particles aggregate in the collagenous connective tissue of humans both extracellularly and by cellular uptake[10,11,17-19]. This process is greatly accelerated in homozygous FH, producing children and young adults with grossly enlarged Achilles tendons, corneal arcus and atherosclerosis.

In 1974 David Cogan wrote "most attempts to correlate the degree of arcus formation with cardiovascular disease have been disappointing. The failure in correlation has been due in part to the inaccessibility of adequate criteria for analogous lipid deposit during life in other tissues of the body[20]." In this study we have used a non-invasive computed score to estimate calcified atherosclerosis in the thoracic aorta and coronary arteries of living patients. We have also calculated a dose-duration measure reflecting the patient's lifelong 'exposure' to the suspected pathogenic agent (i.e. elevated serum cholesterol). These measures were then compared with a measure of the circumferential extent of arcus in the peripheral cornea in an attempt to meet the challenge noted by Dr. Cogan.

## Methods

### Patients

Subjects for this study represented 17 consecutive patients homozygous for FH referred to the Clinical Center of the National Institutes of Health (NIH). Untreated lipoprotein concentrations, electron beam computed tomography and conventional computed tomography results for some of these patients have been reported [21-23]. The Institutional Review Board of the National Heart, Lung and Blood Institute approved this study. Each patient (or for minors, the legal guardians) gave informed consent for the procedures and photographs reported.

### Computed Tomography of Xanthoma and Atherosclerosis

Computed tomography (CT) scans of the Achilles tendon were performed on the GE 9800 scanner (General Electric). Patients were scanned supine with their feet in the neutral position. The displayed field of view was 0.25 m. Contiguous 10-mm thick transaxial scans were obtained through the Achilles tendons from the midcalcaneus level up until the merger with the gastrocnemius muscle. Tendon width was defined as the greatest distance between the most medial and lateral extent of the tendon. The values for Achilles tendon width in this study represent the mean of left and right tendon.

Ultrafast CT for calcific atherosclerosis (CA) was performed and scored according to established methods[22,24]. Tomography was computed for the heart and thoracic aorta. Scans were performed on the Imatron C-100XL CT scanner (Imatron Co.). No intravenous contrast material was used. The coronary artery score represents the sum for left main, left anterior descending, left circumflex, right and posterior descending coronary arteries. The ascending aorta score represents the sum of the aortic root (aorta surrounding the coronary ostia) and the remainder of the ascending aorta. The total score represents the sum of all regions of the thoracic aorta and the coronary arteries.

### Plasma Lipoproteins and Lipids

The fasting plasma concentrations of total cholesterol (TC), LDL and high-density lipoprotein (HDL) were determined by enzymatic assays[25]. All LDL cholesterol values determined at the time of diagnosis were calculated via the Freidwald formula. Study values of lipoproteins were all determined using ultracentrifugation[25]. The cholesterol-year score is an estimate of the lifelong vascular exposure to elevated plasma cholesterol concentrations. The cholesterol year score (CYS) represents the area under the curve for total plasma cholesterol as a function of time.

### Arcus Grading

The photographic slides of both the left and right eye were taken using a 85 mm lens with a Novoflex Bellaco, at 1:1 magnification. The slides of both eyes for each patient were masked with respect to identity and mixed with other slides taken in similar fashion. The slides, as a set, were then given consecutively to 4 judges (one internist and three ophthalmologists). All slides were graded for photo quality and clarity, assessed for presence/absence of corneal arcus, and graded for the extent of arcus using a transparent overlay containing a circle with radius larger than that of the corneo-scleral junction. This circle was divided evenly into eight sectors. Each slide was assessed superiorly and inferiorly (based on the traditional pattern of arcus development) and given a grade of 1 to 4. Grades of 0–8 were assigned for each eye, with each unit representing a sector at least half occupied by corneal arcus. The grades were analyzed for consistency among the different graders. A consensus was reached by the three ophthmologists in cases where all graders did not agree. The grades for both eyes of each patient were then summed and divided by 16 providing a final grade ranging from 0 (no arcus) to 1 (complete circumferential arcus). The grade is a measure of the extent of the 360° of sclero-corneal junction involved in the arcus.

### Statistical Analysis

Statistical analyses were performed using SPSS for Windows, 5.0, SPSSSV for Windows, 6.1 (SPSS Inc.), and Stata, 5.0 (Stata Corp.). For regression analysis the CYS, TC at diagnosis and calcific atherosclerosis scores were transformed in order to obtain a range and distribution better suited for analysis. The base 10 logarithm was taken for all CYS values, TC values at diagnosis, and for CA. Two tailed bivariate correlation coefficients were computed using the Pearson formula. Simple linear regression models were constructed to search for the single most significant predictor for the response of interest. Multiple linear regression models were also constructed to adjust for the confounding effects of age. Comparisons between FH homozygotes and normal control subjects, and comparisons between those patients with and without arcus, and males and females, were done with Student's t tests.

## Results

Table 1 profiles the plasma lipoprotein values both at the time of diagnosis and at the time of this study. The TC and LDL values of these 17 FH homozygotes at diagnosis were significantly (p < .001) higher than those in a sample of the normal population. The HDL values at diagnosis were significantly (p < .001) less than those of the control group. The plasma lipoprotein values at the time of study (TC, LDL, HDL) were also significantly different from the normal values (all p < .001). The cholesterol year scores (CYS) for each of the 17 FH homozygotes were calculated and were markedly increased[23].

**Table 1:** Lipoprotein profiles of 17 FH homozygotes

	Diagnosis				At Study Date				
Patient	TC (mg/dl)	LDL (mg/dl)	HDL (mg/dl)	TC/HDL	TC (mg/dl)	LDL (mg/dl)	HDL (mg/dl)	TC/HDL	CYS (mg*yrs/dl)
1	1200	1150	24	50	1200	NA	24	50	7300
2	1187	1092	40	30	343	288	46	8	27760
3	488	447	29	15	212	150	47	5	7654
4	852	795	24	36	848	709	19	45	11167
5	965	879	29	33	546	494	35	16	12461
6	549	471	33	17	242	193	39	6	11062
7	1277	1153	17	75	184	128	38	5	7366
8	612	572	28	22	264	183	56	5	7650
9	484	405	23	21	484	416	23	21	726
10	740	534	33	22	363	285	61	6	10902
11	1068	1018	24	44	916	804	33	28	2443
12	906	795	32	28	472	424	30	16	9331
13	792	753	22	36	755	701	39	19	4341
14	556	485	40	14	378	335	37	10	5213
15	711	536	56	11	203	104	49	4	8933
16	1198	1148	23	52	845	748	23	37	4599
17	713	650	33	22	482	429	44	11	13219

mean	841	758	30	31	514	399	38	17	8949
sd	261	264	9	16	289	227	12	14	5793

normal mean	159*	96*	49*	3*	159*	96*	49*	3*	--
normal sd	26*	23*	10*	2*	26*	23*	10*	2*	--

*Values from 22 male and 28 female normal volunteers at the Clinical Center of the NIH

NA represents values that are not available

Table 2 lists the age, sex, body mass index, calcific atherosclerosis score, Achilles tendon width, and arcus grade for each individual. The calcific atherosclerosis total score represents the calcific atherosclerosis for the entire thoracic aorta and coronary arteries. The extreme elevation of calcific atherosclerosis above that in a sample of the normal population demonstrates the accelerated vascular disease found in homozygous FH individuals. Of the 17 patients in this study, only those six years or younger in age did not have calcified lesions. These results are consistent with previously reported data showing that in FH homozygotes, atherosclerosis tends to start and be most heavily concentrated in the aortic root[22,26,27].

**Table 2:** Calcific atherosclerosis score, Achilles tendon width, and arcus grade

	Physical Characteristics			Calcific Atherosclerosis Score					
Patient	sex	age (yrs)	BMI (kg/m^2)	Coronary	Ascending (no units)	Descending	Total	Tendon Width (cm)	Arcus Grade (no units)
1	M	6	14.58	0	0	0	0	1.20	0.000
2	F	41	26.76	6376	9909	2570	18855	4.13	1.000
3	M	32	24.21	275	383	0	658	2.20	0.250
4	F	28	23.81	42	1082	733	1857	3.00	0.625
5	F	33	27.10	118	471	0	590	3.10	0.563
6	M	37	25.96	0	258	0	258	1.92	0.641
7	M	21	22.99	11	2407	378	2796	1.60	0.000
8	F	29	20.53	71	248	131	450	2.00	0.250
9	F	1	14.81	0	0	0	0	0.75	0.000
10	F	37	23.65	274	1109	0	1383	2.80	0.500
11	F	3	14.76	0	0	0	0	1.00	0.000
12	F	15	21.77	1	166	88	255	2.00	0.000
13	F	5	19.38	0	0	0	0	1.00	0.000
14	M	14	20.78	0	103	0	103	1.80	0.000
15	M	36	25.89	96	49	0	145	1.90	0.000
16	M	5	17.01	0	0	0	0	1.20	0.094
17	F	39	33.65	11	1720	5	1736	2.50	1.000
mean	--	22	22.21	428	1053	230	1711	2.01	0.290
sd	--	14	5.13	1535	2385	633	4494	0.89	0.360
normal mean+sd	--	--	--	0*	0*	0*	0*	1.18+.12**	--

*Values obtained from 29 control subjects aged 0 to 40

**Values obtained from Liem et al [67].

The Achilles tendon width, measured by CT, was greater (p < .001) than that found in controls. All but the three youngest patients had a tendon width greater than the control mean.

Photographs demonstrating corneal arcus are presented in Fig. 1. The corneal arcus grades had a mean of 0.29 ± .36. There were two patients with total circumferential arcus, 7 patients with partial arcus, and 8 patients with no arcus. Patient 3 and 8 had only inferior arcus in both eyes. Patient 4, 5, 6, 10 and 16 had arcus both inferiorly and superiorly in both eyes but the arcus was not completely circumferential.

**Figure 2:**
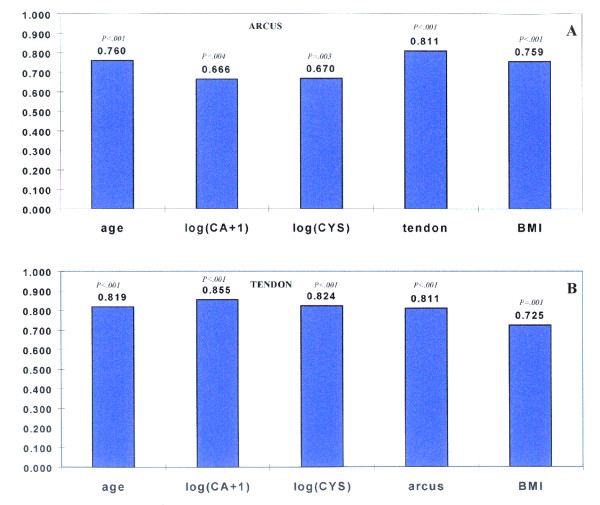
**Correlations**. The correlations of various variables with (A) the extent of arcus, and (B) Achilles tendon width in 17 FH homozygotes. The values on the vertical axis represent Pearson correlation coefficients. The p values represent the significance levels.

Figure 2 shows the bivariate correlation coefficients both for arcus score and for Achilles tendon width. Bar graphs show correlations of various characteristics with (A) arcus grade, and (B) Achilles tendon width in 17 FH homozygotes.

**Figure 3:**
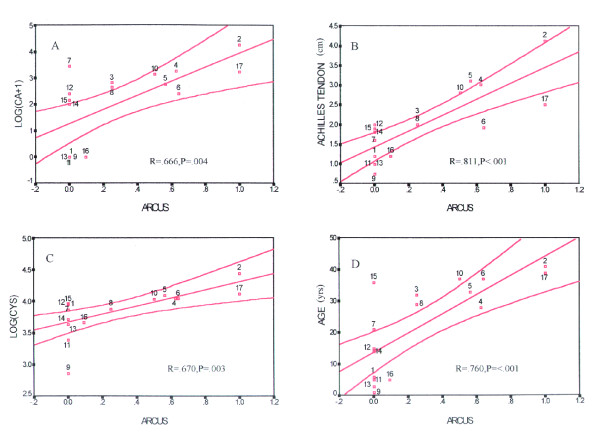
**Best fit plots for arcus score**. Scatter plots with the best-fit lines for (A) log (CA+1), (B) Achilles tendon width, (C) log (CYS), and (D) age, against arcus score in the 17 FH homozygotes. The hourglass shaped boundaries represent 95% confidence intervals.

**Figure 4:**
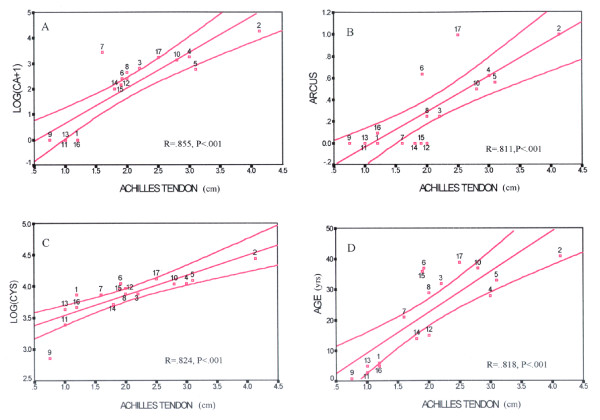
**Best fit plots for Achilles tendon width**. Scatter plots with the best-fit lines for (A) log (CA+1), (B) arcus score, (C) log (CYS), and (D) age, against Achilles tendon width in 17 FH homozygotes. The hourglass shaped boundaries represent the 95% confidence intervals.

Figure 3 illustrates the best-fit lines for log (CA+1), Achilles tendon width, log (CYS), and age plotted against arcus grade. It shows that arcus grade predicts Achilles tendon width best. Figure 4 shows the best-fit lines for the same regressions as in Figure 3 against Achilles tendon width. It shows that Achilles tendon width predicts log (CA+1) best. In both figures the boundaries represent the 95^th ^confidence bands about the fitted lines.

Subgroup analysis indicated no significant differences in any of the study parameters between males and females. However, when patients with arcus (n = 9) and those without (n = 8) were compared, the Achilles tendon width was significantly greater (p = .004) for those with arcus (mean 2.54 ± 0.84) than those without (mean 1.41 ± 0.48). In addition, log (CA+1) was significantly greater (p = .035) in those with arcus (mean 2.73 ± 1.15) then those without (mean 1.26 ± 1.41). The CYS was significantly higher (p = .028) for those patients with arcus (mean 11830 ± 6569) than for those without (mean 5707 ± 3080). The age was significantly higher (p = .004) for those with arcus (mean 31 ± 11) than for those without arcus (mean 13 ± 12).

## Discussion

Attempts have been made to correlate corneal arcus with factors as diverse as alcoholism, race, and exposure to atomic bomb detonation [28-31]. The current study was designed to assess the correlation of arcus with tendinous deposits, with atherosclerotic deposits, and a score for an estimate of lipid exposure over a lifetime. The accelerated course of lipid deposition in the patients in this study presents an opportunity to evaluate these relationships. We did find a correlation between corneal arcus and calcified atherosclerosis in these patients, however arcus did not prove to be as highly correlated with atherosclerosis as tendon xanthoma.

The lipid deposition process across tissues is presumably similar enough to produce the correlations found, and this is the reason why one might consider looking to the eye for some clues about what is happening in the coronaries. However, the lipid deposition process across tissues is also subject to differences. In addition, the means of measuring these deposits is also an important consideration.

The measure of calcific atherosclerosis was selected as the endpoint to quantitate the extent of atherosclerosis. This is, however, a conservative estimate of the amount of atherosclerosis in the coronary arteries and thoracic aorta since CT only detects those plaques that have become calcified and does not measure the extent of fatty streaks or "soft" atheroma[22]. This underestimate may be greater in children than older adults. Therefore the correlation here is between corneal arcus and advanced atherosclerotic lesions.

Some authors in the past have noted the lack of correlation between plasma lipid levels and corneal arcus [32-34]. Suspicion that the varying roles of dose-dependency and duration-dependency are complicating this relationship have been suggested[35,36]. The cholesterol year score provides a dose-duration measure. Such measures are proving important in explaining the loss of predictive value in certain risk factor analysis based on a single measure of dose[37]. The arcus score correlated with log CYS (r = .670, P = .003), but not with the log of total cholesterol at the study date (r = -.104, P = .691).

The correlation of calcific atherosclerosis with other variables was paralleled by the correlation between the calcific atherosclerosis score and age. In developing a regression model for calcific atherosclerosis, a combination of age and Achilles tendon width provided the best fit. The best single predictor of the extent of calcific atherosclerosis was Achilles tendon, which can be used in a quadratic equation to predict the calcific atherosclerosis in these patients (data not shown). Taking the derivative of the quadratic equation for CA as a function of Achilles tendon width produces a linear function representing the rate of change in CA as tendon changes. This derivative indicates that as a tendon of smaller or normal width changes, the resulting increase in CA is larger than for the same increment of change in a larger tendon, namely a tendon already laden with lipid. This implication and the strong relation between CA and Achilles tendon, stress the important diagnostic value of tendinous xanthoma for hyperlipidemia and for assessing vascular health in FH.

The high correlation of Achilles tendon with arcus and calcific atherosclerosis, persisting after adjustment for age, may be due to the similarities and differences in the underlying mechanisms of the three types of deposition. All these tissues consist of collagenous connective tissue with glycoaminoglycans embedded in the matrix. The predominant lipid in these deposits is cholesteryl ester, although unesterified cholesterol is also present. A major difference between the cornea, on the one hand, and arterial wall and tendon[38], on the other, is that macrophages do not infiltrate the cornea. The respective presence and absence of macrophage activity may alter the type of lipid in these deposits and their distribution[17,39]. Arcus is only the macroscopic sign of lipid deposition, and amounts of lipid can be deposited in the perivascular ground substance of the eye without producing a visible arcus[17,40,41]. The overall distribution of lipid and the structure and size of the lipid particles may influence the threshold of deposition for arcus visibility. These and other differences in corneal deposition may explain why Achilles tendon is a better predictor of calcific atherosclerosis than arcus.

Even in the individuals in this study, who have sustained high LDL cholesterol levels, the deposits of lipid that form arcus stay to the periphery of the cornea. Work by Phillips, et al[42], Ashraf, et al[43] and Crispin[4] suggest some reasons for the macroscopic appearance of corneal arcus. The cornea has a temperature gradient which can effect lipid deposition. It also has a gradient regarding how dense the collagen fibers of the cornea are packed and this effects what size of particle can move towards the center. Furthermore, the cornea is normally avascular, with only the periphery close to limbal capillary beds, which are the source of the lipid in arcus[44,45]. It may be that the cornea protects itself from lipid deposition both by filtering out the large lipoprotein particles in the periphery and by relying on nascent HDL, which is a particle small enough to move across the cornea, for uptake of excess cholesterol. The patients in this study not only have elevated LDL, but tend to have lower than normal HDL. Others have suggested a relationship between low HDL levels and corneal arcus[46,47]. Patients suffering from classical LCAT deficiency or from other genetic errors disrupting the HDL reverse transport pathway (such as Tangier's disease or Fish Eye Disease) suffer from opacities of the central cornea.

The LDL particles that presumably transport and deposit lipid in the cornea may undergo oxidative and other changes that make arcus more likely, and patients with LDL receptor defects, such as the ones in this study, are known to have LDL particles that circulate longer and probably undergo changes that make them more atherogenic. Such changes to LDL may also be important in generating corneal arcus[19,28,36,48].

The number of patients with measured arcus of zero was significant. As noted above, 8 of the 17 patients have no arcus, and these 8 are significantly younger as a group (mean age 13 ± 12) than those with arcus (mean age 31 ± 11). The data on these 17 patients was collected over several decades and in some cases is ongoing. With the advent of newer therapies such as LDL removal by plasmapheresis, younger patients were in many cases receiving more aggressive therapy at younger ages[49]. One of the patients also underwent a liver transplant in the second decade of his life. This patient's cornea was photographed after the transplant and shows no arcus. No photo exists of this patient's cornea before transplant, but anecdotal reports by physicians following the patient pre-transplant claim some arcus was beginning to become visible. These therapeutic interventions may effect lipid deposition in the eye differently than those in tendons or those represented as calcified atherosclerotic plaques. On the other hand, the very youngest patients would not have received treatment for very long. The age of onset of visible arcus may be somewhat delayed for patients whose therapy was more effective, began earlier, and was carried out for several years. Thus, even though CYS was used to mitigate some of these issues, differences in therapeutic intervention may still be further confounding attempts to correlate relationships between various kinds of lipid deposits in these patients.

We have studied patients with a rare inborn error of metabolism causing accelerated deposition of lipid in tissues. However, a review of the literature shows that corneal arcus has also been correlated with the incidence of hypercholesterolemia and the incidence of coronary heart disease in larger populations [50-66]. Based on this literature, in patients younger than 50 with corneal arcus, there is an increased risk of coronary heart disease and a high probability that they may have some variety of hypercholesterolemia. Furthermore, detecting corneal arcus and assessing its circumferential extent (on a scale from 1–8) is an assessment that might be made quickly on physical exam.

## Conclusion

In the 17 patients considered in this study, the presence of corneal arcus was correlated with tissue cholesterol deposition detectable by computed tomography. The patients in our study with more extensive circumferential arcus tended to have more severe atherosclerosis. The underlying mechanisms of lipid deposition are probably related (thus there was some correlation) but not similar enough to provide fine-grained prediction. Achilles tendon xanthoma was a better indicator of the extent of atherosclerosis in these patients, and it is an indicator of FH at any age, and can often be detected by palpation. Considering this study and the growing literature, corneal arcus in people less than 50 years of age should be regarded as an indicator of hyperlipidemia. Therefore as an easily observed clinical finding, the presence of either xanthoma or arcus should raise suspicion for hypercholesterolemia as well as the presence of atherosclerosis. Further study of these relationships is warranted.

## Abbreviations

CA-calcific atherosclerosis; CT-computed tomography; CYS-cholesterol year score; FH-familial hypercholesterolemia; HDL-high density lipoprotein; LDL-low density lipoprotein; TC-total cholesterol

## Competing interests

The author(s) declare that they have no competing interests.

## Authors' contributions

LAZ designed the study, performed all statistical analysis, drafted and edited the manuscript. JMH participated (over many years) in producing the data, assisted with the design of the study, participated in grading the extent of arcus, and reviewed the final manuscript.

## References

[B1] Virchow R (1852). Ueber perenchymatose Entzundung. Archiv für pathologische Anatomie und Physiologie und für klinische Medicin.

[B2] Osler W (1892). The principles and practice of medicine: designed for the use of practitioners and students of medicine.

[B3] Fernandez A, Sorokin A, Thompson PD (2007). Corneal arcus as coronary artery disease risk factor. Atherosclerosis.

[B4] Crispin S (2002). Ocular lipid deposition and hyperlipoproteinaemia. Prog Retin Eye Res.

[B5] Goldstein JL, Hobbs HH, Brown MS, Scriver CR, Beaudet AL, Sly WS, Valle D (1995). Familial Hypercholesterolemia. The Metabolic and Molecular Bases of Inherited Diseases.

[B6] Hoeg JM (1994). Familial hypercholesterolemia. What the zebra can teach us about the horse. Jama.

[B7] Goldstein JL, Brown MS (1977). The low-density lipoprotein pathway and its relation to atherosclerosis. Annu Rev Biochem.

[B8] Fredrickson DS, Lees RS, Stanbury JB, Wyngaarden JB, Fredrickson DS (1966). Familial Hyperlipoproteinemia. The metabolic basis of inherited disease.

[B9] Brewer HBJ, Santamarina-Fojo SM, Shamburek R, Fuster V, Topol EJ, Nabel E (2005). Genetic Dyslipoproteinemias. Atherothrombosis and coronary artery disease.

[B10] Xu Q, Fitridge R, Thompson M (2007). Atherosclerosis. Mechanisms of vascular disease: a textbook for vascular surgeons.

[B11] Lusis AJ (2000). Atherosclerosis. Nature.

[B12] LaRosa JC, Sidawy AN, Sumpio BE, DePalma RG (1997). Plasma Lipoproteins and Vascular Disease. The Basic Science of Vascular Disease.

[B13] Kane JP, Fuster V, Topol EJ, Nabel E (2005). Structure and Function of the Plasma Lipoproteins and Their Receptors. Atherothrombosis and Coronary Artery Disease.

[B14] Adams CW, Bayliss OB, Baker RW, Abdulla YH, Hunter-Craig CJ (1974). Lipid deposits in ageing human arteries, tendons and fascia. Atherosclerosis.

[B15] Parker F, Short JM (1970). Xanthomatosis associated with hyperlipoproteinemia. J Invest Dermatol.

[B16] Sheraidah GA, Winder AF, Fielder AR (1981). Lipid-protein constituents of human corneal arcus. Atherosclerosis.

[B17] Gaynor PM, Zhang WY, Salehizadeh B, Pettiford B, Kruth HS (1996). Cholesterol accumulation in human cornea: evidence that extracellular cholesteryl ester-rich lipid particles deposit independently of foam cells. J Lipid Res.

[B18] Hansson GK (2005). Inflammation, atherosclerosis, and coronary artery disease. N Engl J Med.

[B19] Steinberg D, Parthasarathy S, Carew TE, Khoo JC, Witztum JL (1989). Beyond cholesterol. Modifications of low-density lipoprotein that increase its atherogenicity. N Engl J Med.

[B20] Cogan DG (1974). Editorial: The corneal arcus. N Engl J Med.

[B21] Dugi KA, Feuerstein IM, Hill S, Shih J, Santamarina-Fojo S, Brewer HB, Hoeg JM (1997). Lipoprotein lipase correlates positively and hepatic lipase inversely with calcific atherosclerosis in homozygous familial hypercholesterolemia. Arterioscler Thromb Vasc Biol.

[B22] Hoeg JM, Feuerstein IM, Tucker EE (1994). Detection and quantitation of calcific atherosclerosis by ultrafast computed tomography in children and young adults with homozygous familial hypercholesterolemia. Arterioscler Thromb.

[B23] Schmidt HH, Hill S, Makariou EV, Feuerstein IM, Dugi KA, Hoeg JM (1996). Relation of cholesterol-year score to severity of calcific atherosclerosis and tissue deposition in homozygous familial hypercholesterolemia. Am J Cardiol.

[B24] Agatston AS, Janowitz WR, Hildner FJ, Zusmer NR, Viamonte M, Detrano R (1990). Quantification of coronary artery calcium using ultrafast computed tomography. J Am Coll Cardiol.

[B25] Hoeg JM, Maher MB, Bou E, Zech LA, Bailey KR, Gregg RE, Sprecher DL, Susser JK, Pikus AM, Brewer HB (1984). Normalization of plasma lipoprotein concentrations in patients with type II hyperlipoproteinemia by combined use of neomycin and niacin. Circulation.

[B26] Sprecher DL, Schaefer EJ, Kent KM, Gregg RE, Zech LA, Hoeg JM, McManus B, Roberts WC, Brewer HB (1984). Cardiovascular features of homozygous familial hypercholesterolemia: analysis of 16 patients. Am J Cardiol.

[B27] Buja LM, Kovanen PT, Bilheimer DW (1979). Cellular pathology of homozygous familial hypercholesterolemia. Am J Pathol.

[B28] Ewing JA, Rouse BA (1980). Corneal arcus as a sign of possible alcoholism. Alcohol Clin Exp Res.

[B29] Hirose I (1962). [Relationship between the occurrence of arcus senilis of the cornea and condition subjected to the exposure of an atomic bomb.]. Nippon Ganka Kiyo.

[B30] Macaraeg PV, Lasagna L, Snyderb (1968). Arcus not so senilis. Ann Intern Med.

[B31] Tschetter RT (1966). Arcus senilis: Its Relationship to Serum Lipids in the Negro Male. Arch Ophthalmol.

[B32] Lindholm H (1960). Arcus lipoides corneae and arteriosclerosis. Acta Med Scand.

[B33] McAndrew GM, Ogston D (1965). Arcus senilis and coronary artery disease. Am Heart J.

[B34] Hickey N, Maurer B, Mulcahy R (1970). Arcus senilis: its relation to certain attributes and risk factors in patients with coronary heart disease. Br Heart J.

[B35] Winder AF (1983). Relationship between corneal arcus and hyperlipidaemia is clarified by studies in familial hypercholesterolaemia. Br J Ophthalmol.

[B36] Rouhiainen P, Salonen R, Rouhiainen H, Salonen JT (1993). Association of corneal arcus with ultrasonographically assessed arterial wall thickness and serum lipids. Cornea.

[B37] Wilson PW, Hoeg JM, D'Agostino RB, Silbershatz H, Belanger AM, Poehlmann H, O'Leary D, Wolf PA (1997). Cumulative effects of high cholesterol levels, high blood pressure, and cigarette smoking on carotid stenosis. N Engl J Med.

[B38] Kruth HS (1985). Lipid deposition in human tendon xanthoma. Am J Pathol.

[B39] Gerrity RG (1981). The role of the monocyte in atherogenesis: I. Transition of blood-borne monocytes into foam cells in fatty lesions. Am J Pathol.

[B40] Cogan DG, Kuwabara T (1959). Arcus senilis; its pathology and histochemistry. AMA Arch Ophthalmol.

[B41] Walton KW (1973). Studies on the pathogenesis of corneal arcus formation. I. The human corneal arcus and its relation to atherosclerosis as studied by immunofluorescence. J Pathol.

[B42] Phillips CI, Tsukahara S, Gore SM (1990). Corneal arcus: some morphology and applied pathophysiology. Jpn J Ophthalmol.

[B43] Ashraf F, Cogan DG, Kruth HS (1993). Apolipoprotein A-I and B distribution in the human cornea. Invest Ophthalmol Vis Sci.

[B44] Fielder AR, Winder AF, Sheraidah GA, Cooke ED (1981). Problems with corneal arcus. Trans Ophthalmol Soc U K.

[B45] Barouch FC, Colby K, Foster CS, Azar DT, Dohlman CH (2005). Corneal and Conjuctival Degenerations. Smolin and Thoft's The Cornea.

[B46] Meyer D, Liebenberg PH, Maritz FJ (2004). Serum lipid parameters and the prevalence of corneal arcus in a dyslipidaemic patient population. Cardiovasc J S Afr.

[B47] Hoogerbrugge N, Happee C, van Domburg R, Poldermans D, van den Brand MJ (1999). Corneal arcus: indicator for severity of coronary atherosclerosis?. Neth J Med.

[B48] Mosinger BJ (1997). Human low-density lipoproteins: oxidative modification and its relation to age, gender, menopausal status and cholesterol concentrations. Eur J Clin Chem Clin Biochem.

[B49] Yokoyama S, Hayashi R, Satani M, Yamamoto A (1985). Selective removal of low density lipoprotein by plasmapheresis in familial hypercholesterolemia. Arteriosclerosis.

[B50] Rosenman RH, Brand RJ, Sholtz RI, Jenkins CD (1974). Relation of corneal arcus to cardiovascular risk factors and the incidence of coronary disease. N Engl J Med.

[B51] Klein B, Klein R, Haseman J, Maready J, Hames C (1975). Corneal arcus and cardiovascular disease in Evans County, Georgia. Arch Intern Med.

[B52] Chambless LE, Fuchs FD, Linn S, Kritchevsky SB, Larosa JC, Segal P, Rifkind BM (1990). The association of corneal arcus with coronary heart disease and cardiovascular disease mortality in the Lipid Research Clinics Mortality Follow-up Study. Am J Public Health.

[B53] Hughes K, Lun KC, Sothy SP, Thai AC, Leong WP, Yeo PB (1992). Corneal arcus and cardiovascular risk factors in Asians in Singapore. Int J Epidemiol.

[B54] (1991). Risk of fatal coronary heart disease in familial hypercholesterolaemia. Scientific Steering Committee on behalf of the Simon Broome Register Group. Bmj.

[B55] Parwaresch MR, Haacke H, Mader C, Godt C (1976). Arcus lipoides corneae und hyperlipoproteinamie. Klinische Wochenschrift.

[B56] Jaeger W, Eisenhauer GG (1977). [The diagnostic value of corneal arcus as symptom of hyperlipoproteinemia (author's transl)]. Klin Monatsbl Augenheilkd.

[B57] Segal P, Insull W, Chambless LE, Stinnett S, LaRosa JC, Weissfeld L, Halfon S, Kwiterovitch PO, Little JA (1986). The association of dyslipoproteinemia with corneal arcus and xanthelasma. The Lipid Research Clinics Program Prevalence Study. Circulation.

[B58] Nishimoto JH, Townsend JC, Selvin GJ, De Land PN (1990). Corneal arcus as an indicator of hypercholesterolemia. J Am Optom Assoc.

[B59] Schneider T, Ulbig M (1991). [Diagnostic and functional significance of arcus lipoides in hypercholesterolemia]. Fortschr Ophthalmol.

[B60] Winder AF, Jolleys JC, Day LB, Butowski PF (1998). Corneal arcus, case finding and definition of individual clinical risk in heterozygous familial hypercholesterolaemia. Clin Genet.

[B61] Lertchavanakul A, Laksanaphuk P, Tomtitchong T (2002). Corneal arcus associated with dyslipidemia. J Med Assoc Thai.

[B62] Slack J (1969). Risks of ischaemic heart-disease in familial hyperlipoproteinaemic states. Lancet.

[B63] Stone NJ, Levy RI, Fredrickson DS, Verter J (1974). Coronary artery disease in 116 kindred with familial type II hyperlipoproteinemia. Circulation.

[B64] Gagne C, Moorjani S, Brun D, Toussaint M, Lupien PJ (1979). Heterozygous familial hypercholesterolemia. Relationship between plasma lipids, lipoproteins, clinical manifestations and ischaemic heart disease in men and women. Atherosclerosis.

[B65] Goldstein JL, Hazzard WR, Schrott HG, Bierman EL, Motulsky AG (1972). Genetics of hyperlipidemia in coronary heart disease. Trans Assoc Am Physicians.

[B66] Hazzard WR, Goldstein JL, Schrott MG, Motulsky AG, Bierman EL (1973). Hyperlipidemia in coronary heart disease. 3. Evaluation of lipoprotein phenotypes of 156 genetically defined survivors of myocardial infarction. J Clin Invest.

[B67] Liem MS, Leuven JA, Bloem JL, Schipper J (1992). Magnetic resonance imaging of Achilles tendon xanthomas in familial hypercholesterolemia. Skeletal Radiol.

